# Dry preservation of *Toxocara vitulorum* by plastination technique

**DOI:** 10.14202/vetworld.2019.1428-1433

**Published:** 2019-09

**Authors:** Niranjan Kumar, Jayesh B. Solanki, Prabhakar Shil, Dharmesh C. Patel, Ramasamy Meneka, Shailendra Chaurasia

**Affiliations:** 1 Department of Veterinary Parasitology, College of Veterinary Science and Animal Husbandry, Navsari Agricultural University, Navsari, Gujarat, India; 2 Department of Veterinary Anatomy, College of Veterinary Science and Animal Husbandry, Navsari Agricultural University, Navsari, Gujarat, India

**Keywords:** melamine, plastination, preservation, *Toxocara vitulorum*

## Abstract

**Background and Aim:**

The most widely adopted technique to preserve the gross specimen of the parasite is immersions and storage in liquid preservatives. The present study aimed to describe the dry method of the preservation of *Toxocara* *vitulorum* using plastination technique.

**Materials and Methods:**

Acetone dehydrated parasites were incubated at −20°C for 1 month in five different plastination solutions, prepared by mixing melamine and turpentine oil with clove oil (MTCl)/chloroform (MTC)/isopropanol (MTI)/benzene (MTB)/xylene (MTX) in 1:1:1 ratio to infiltrate the polymer. Technical personnel was asked to assign weekly score for dryness, stickiness, shrinkage, glossiness, flexibility, and odor of the prepared model on a 5-point scale.

**Results:**

Overall, the plastinated parasites were dry, non-sticky, glossy, odorless, chemical-free, harmless, to some extent flexible, with detectable morphological structure including natural form but lost their natural color, and cuticle became translucent. A varying level of shrinkage was noted in all types of plastinated model, but it was least in MTCl model. One month post-plastination, the mean evaluation score for glossiness was maximum in the parasite plastinated in MTCl solution (4.50±0.17), followed by MTC (3.72±0.32), MTX (3.56±0.38), MTB (2.83±0.37), and MTI (2.31±0.33). Likewise, for flexibility, the score was maximum in the parasite plastinated in MTCl solution (4.36±0.16), followed by MTB (3.11±0.14), MTC (2.94±0.41), MTX (2.75±0.41), and MTI (1.97±0.28). The degree of dryness, stickiness, and odor of the prepared model varies non-significantly (p>0.05) with the polymer mixtures. Maximum shrinkage percentage in terms of length and width was 4.24% and 50%, respectively, in the parasites plastinated in MTB solution. Shrinkage percentage was minimal (1.81% in length and 25% in width) in the MTCl plastinated parasites. Shrinkage percentage in terms of dimension was statistically non-significant among the different polymer solutions. Plastinated models withstand the process of microbial decomposition. There were 5 and 11 odd points in favor of plastination and formalin preservation technique, respectively.

**Conclusion:**

The prepared *T*. *vitulorum* model in MTCl can be used as an adjunct to the parasite preserve in 10% formalin solution. The plastination technique can be used as an alternative method of liquid preservation.

## Introduction

The roundworm, *Toxocara* *vitulorum* (synonym *Neoascari*s *vitulorum*) occurs in the small intestine of ruminants all around the world [1]. The main hosts of *T*. *vitulorum* are young cattle and Indian buffalo [2], where it causes significant economic loss in terms of morbidity and mortality of the animals [3].

Educating the public, including students using the gross specimen of the parasite can become an important step to meet the sustainable level of control against the parasites [4]. The most widely adopted technique to preserve the gross specimen of the parasites in the academic institutions is immersion and storage in 10% neutral buffered formalin, often used to say formalin (contain approximately 4% formaldehyde) or 70% ethyl alcohol [5]. The stored biological specimens in these liquids are inherited with limitation of wet, noxious odor, hazardous to the handlers, difficult to transport, and distorted morphological features [6,7]. The handlers can develop disease of eye, skin, respiratory and nervous system, and sometimes development of allergy and cancer [8,9]. Alternatively, plastination is the most recent and innovative technique of dry preservation of the biological samples. The procedure consists of slow replacement of body fluids and adipose tissue by a curable polymer under defined conditions [10]. Plastinated specimens are clean, dry, odorless, resistant, durable, and free of toxic chemicals, thus can be handled without any precautionary measures, i.e., use of gloves or mask [11,12]. Further plastinated tissues minimize the daily exposure of learner and technical staff to a hazardous substance like formaldehyde [9]. The technique gain popularity among the anatomist, while very few parasitologists have reported its practical utility in preserving the parasites [13-17]. Melamine is one of the important polymers for the plastination technique [17,18]. Melamine, a nitrogen-rich heterocyclic triazine, is mainly used to manufacture the plastics, coatings materials, filters, adhesives, dishware, and kitchenware [19,20].

The present study aimed to describe the plastination of *Toxocara* (*Neoascaris*) *vitulorum* by infiltrating melamine polymer in the parasite tissue. For this, five polymer solutions of melamine and turpentine oil with chloroform /clove oil /isopropanol /benzene /xylene were used. A comparative study for dryness, stickiness, shrinkage, glossiness, flexibility, and odor was done for all five plastinated specimens.

## Materials and Methods

### Ethical approval

The ethical committee approval was not required, as the study was conducted using the gross specimen of the roundworm stored in 10% formalin in the departmental museum.

### Parasite specimens

The gross specimens of *Toxocara* (*Neoascaris*) *vitulorum* stored in 10% formalin in the museum of the Department of Veterinary Parasitology, College of Veterinary Science and Animal Husbandry, Navsari Agricultural University, Navsari-396 450, Gujarat, India, were subjected for the plastination in different polymer solutions. To measure the extent of shrinkage, the dimension of the specimens was taken before and after impregnation of the polymer.

### Plastinating materials

The melamine polymer and touchwood of Asian Paints along with turpentine and clove oil were procured from the local market. The acetone, xylene, isopropanol, benzene, and chloroform were of Merck make.

### Plastination technique

In the present investigation, plastination technique was performed as per the method described by Kumar *et al*. [17] with certain modification. The worms were washed vigorously for 24 h in the cold water (5°C) to remove the formalin solution [14]. The specimens were dehydrated at −20°C in the three changes of absolute acetone at weekly interval. Five polymer solutions were prepared by mixing melamine, turpentine oil and chloroform (MTC)/clove oil (MTCl)/isopropanol (MTI)/benzene (MTB)/xylene (MTX) in 1:1:1 ratio. The dehydrated *T*. *vitulorum* was dipped in these polymer solutions. The parasite-polymer solution was incubated at −20°C for 30 days with gentle stirring at regular interval. Following forced impregnation, the specimens were removed from the plastinating solution and kept in a Petri dish for 2-3 days to drain out the excess polymer. Each specimen was brushed with colorless varnish to impart glossy appearance. The plastinated *T*. *vitulorum* models were evaluated for dryness, stickiness, shrinkage, glossiness, flexibility, and odor.

### Evaluation of make quality

Nine technical staffs had assigned a score of dryness, stickiness, shrinkage, glossiness, flexibility, and odor of the prepared model on a 5-point scale at weekly interval. The high score was in favor of the above-listed qualitative parameters. The best plastinated model was with maximum score for dryness, glossiness, and flexibility, while minimum score for stickiness, shrinkage, and odor.

### Evaluation of keeping quality

The study area is 12 km away from the Arabian Sea, and the past 10 years (2009-2018) annual humidity range was 40.2±1.74% (January or February) to 86.1±0.53% (July or August). The high humid environment can accelerate the process of microbial decomposition of the biological samples. Stability of the plastinated model was observed for 2 years at room temperature in the environment. The effect of water on the plastinated model was also observed by dipping them inside the water for 30 min.

### Comparison between plastination and formalin preservation

Qualitative parameters of dryness, flexibility, non-fragility, glossiness, no shrinkage, unstickiness, non-pungent, natural color, transparent cuticle, gross morphology, touch, easy transportation, one time use of preservative, stability in the environment, and no sealed container requirement were answered in either “Yes” or “No.” For comparative study of dry and liquid preservation, “Yes” was assigned “10 points” and “No” with “0 point.”

### Statistical analysis

Results were compiled systematically, and data were analyzed using IBM SPSS Statistics 20.00 of Windows (SPSS Inc., Chicago, USA) to calculate the statistical significance. p>0.05 was considered as non-significant.

## Results

**Figure-1:**
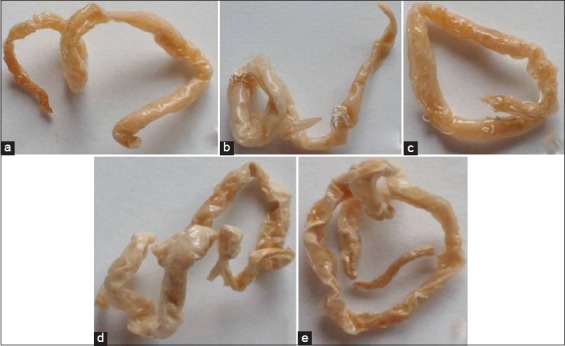
*Toxocara* *vitulorum* plastinated in melamine and turpentine oil with clove oil (MTCl) (a), chloroform (MTC) (b), xylene (MTX) (c), benzene (MTB) (d), and isopropanol (MTI) (e) polymer solutions.

Overall, the plastinated *T*. *vitulorum* parasites were dry, non-sticky, glossy, odorless, chemical-free, harmless, to some extent flexible, with detectable morphological structure including natural form but lost their natural color, and cuticle became translucent (Figure-1a-e). The weekly assigned score of dryness, stickiness, shrinkage, glossiness, flexibility, and odor of the prepared model on a 5-point scale by technical personnel is shown in Table-1. Although almost all plastinated parasites in different polymer solutions had considerable level of dryness, the parasites plastinated in MTB or MTI solution had the highest score of dryness (Table-1 and Figure-1d-e). Corresponding to dryness, maximum fragility was observed in the MTI and MTB plastinated parasites. At all points of time, the score of stickiness was maximum for the MTCl plastinated parasites. A varying level of shrinkage was noted in all types of plastinated model, but it was least in MTCl model (Figure-1a-e). One month post-plastination, the mean evaluation score of glossiness was maximum in the parasite plastinated in MTCl solution (4.50±0.17), followed by MTC (3.72±0.32), MTX (3.56±0.38), MTB (2.83±0.37), and MTI (2.31±0.33) (Table-1). Likewise, for flexibility, the score was maximum in the parasite plastinated in MTCl solution (4.36±0.16), followed by MTB (3.11±0.14), MTC (2.94±0.41), MTX (2.75±0.41), and MTI (1.97±0.28) (Table-1). The parasites plastinated in different polymer solutions lacked pungent odor. The MTCl model initially had the smell of clove oil, but with the pace of time, the smell becomes undetectable. The degree of dryness, stickiness, and odor of the prepared model varies non-significantly with the polymer mixtures.

**Table 1:** Average score of the qualitative parameters of the plastinated model *Toxocara* (*Neoascaris*) *vitulorum* on 5-point scale.

Parameters	Model	1^st^ week		2^nd^ week		3^rd^ week		4^th^ week	
			
		Mean±SE	p-value	Mean±SE	p-value	Mean±SE	p-value	Mean±SE	p-value
Dryness	MTX	4.47±0.17	0.107	4.39±0.16	0.003	4.28±0.22	0.470	4.14±0.26	0.564
	MTB	3.89±0.39		4.22±0.22		4.33±0.24		4.44±0.24	
	MTI	3.67±0.50		4.11±0.31		4.17±0.26		4.50±0.16	
	MTCl	3.11±0.35		2.83±0.33		3.72±0.32		3.94±0.34	
	MTC	4.08±0.27		3.81±0.33		4.22±0.22		4.25±0.28	
Stickiness	MTX	2.44±0.38	0.000	2.11±0.42	0.008	1.56±0.18	0.021	1.11±0.11	0.527
	MTB	2.06±0.36		1.89±0.42		1.33±0.17		1.44±0.29	
	MTI	1.33±0.17		2.11±0.42		1.67±0.29		1.44±0.18	
	MTCl	4.67±0.17		3.94±0.34		2.56±0.38		1.67±0.37	
	MTC	3.83±0.26		2.61±0.45		1.78±0.22		1.22±0.15	
Shrinkage	MTX	2.56±0.29	0.00	3.11±0.31	0.00	3.11±0.31	0.00	3.11±0.26	0.001
	MTB	3.67±0.14		4.44±0.18		4.11±0.26		3.89±0.35	
	MTI	4.47±0.33		4.11±0.35		4.33±0.29		3.11±0.45	
	MTCl	1.44±0.18		2.11±0.356		2.00±0.24		1.67±0.17	
	MTC	2.00±0.24		3.50±0.29		3.33±0.17		3.0±0.33	
Glossiness	MTX	4.19±0.20	0.00	4.39±0.20	0.00	4.28±0.32	0.007	3.56±0.38	0.00
	MTB	3.19±0.27		3.28±0.25		3.11±0.35		2.83±0.37	
	MTI	2.17±0.33		2.22±0.32		2.72±0.30		2.31±0.33	
	MTCl	4.03±0.44		4.58±0.17		4.17±0.31		4.50±0.17	
	MTC	4.28±0.22		4.17±0.17		3.72±0.37		3.72±0.32	
Flexibility	MTX	4.22±0.22	0.00	3.33±0.22	0.00	2.97±0.360	0.00	2.75±0.41	0.00
	MTB	2.94±0.37		2.61±0.50		1.78±0.28		3.11±0.14	
	MTI	1.69±0.31		1.56±0.24		1.44±0.246		1.97±0.28	
	MTCl	4.33±0.25		4.72±0.147		4.67±0.17		4.36±0.16	
	MTC	3.80±0.23		3.61±0.29		2.72±0.30		2.94±0.41	
Odor	MTX	3.58±0.38	0.378	4.00±0.29	0.121	3.14±0.43	0.393	3.56±0.50	0.812
	MTB	3.33±0.41		3.22±0.46		3.47±0.34		3.31±0.55	
	MTI	3.44±0.44		3.22±0.43		3.28±0.42		3.42±0.48	
	MTCl	4.08±0.29		4.33±0.22		4.11±0.25		4.08±0.34	
	MTC	4.14±0.20		3.92±0.31		3.47±0.34		3.58±0.48	

MTX=Melamine and turpentine oil with xylene, MTC=Melamine and turpentine oil with chloroform, MTI=Melamine and turpentine oil with isopropanol, MTB=Melamine and turpentine oil with benzene, MTCl=Melamine and turpentine oil with clove oil, SE=Standard error

The plastinated worms were up to 16.5 cm×8 mm, translucent cuticle; body did not taper toward the extremities (Figure-1). Maximum shrinkage percentage in terms of length and width was 4.24% and 50%, respectively, in the parasites plastinated in MTB solution. Shrinkage percentage was minimal (1.81% in length and 25% in width) in the MTCl plastinated parasites. Shrinkage percentage in terms of dimension was statistically non-significant among the polymer solutions used.

The hydrophobic feature of the plastinated model was observed, as it dried quickly when taken out from the water tank. The plastinated models withstand the process of microbial decomposition and remain intact in the environment of high level of humidity and precipitation until the drafting of the manuscript.

When a comparison was made in terms of dryness, flexibility, non-fragility, glossiness, no shrinkage, unstickiness, non-pungency, natural color, transparent cuticle, gross morphology, hand touch, easy to transport, one time use of preservative, stability in environment, and no sealed container requirement, there were 5 odd points with score of 100 in favor of the plastination techniques, while it was 11 odd points with score of 40 for formalin preservation technique (Table-2).

**Table 2:** Comparison between plastination (dry) and formalin (liquid) preservation of *Toxocara* (*Neoascaris*) *vitulorum.*

Preservation	Qualitative parameters															**Total score

	Dry	Flexible	Non-fragile	Glossy	No shrinkage	Unsticky	Non- pungent	Natural color	Transparent cuticle	Gross morphology	Touch	Transportation easy	1 time use of preservative	Stability in environment	No sealed container requirement	
Plastination	Yes	*No	No	Yes	No	Yes	Yes	No	No	Yes	Yes	Yes	Yes	Yes	Yes	100
Formalin	No	Yes	No	No	No	Yes	No	No	No	Yes	No	No	No	Yes	No	40

* No to minimal (depending on the polymer solution).

** Yes=10 and No=0

## Discussion

Plastination technique was originally described by Gunther von Hagens, in 1977, to preserve the biological specimens [10]. The technique rapidly gained its popularity in the medical world, especially among the anatomist [11]. The technique widely used by the anatomist to preserve the hard tissue like bone [12]. Gradually, the scientists were making their efforts to preserve the hard or soft tissue using this technique [21]. It is the need of the time to have an alternative method of preservation of the biological samples. Being a dry method of preservation, the plastination technique can be an excellent alternative with higher health and safety regulations, as it lowers the risk of undue exposure to the formaldehyde [17,18]. Furthermore, plastinated specimens are easy to carry, palpable, with clearly visible structure, and can be stored for an infinite period at room temperature [22].

For the first time, Asadi and Mahmodzaeh [13] had used S10 plastination technique to plastinate the *Ascaris lumbricoide*s. Since then, scientists did necessary changes in the plastination protocol to develop ideal plastinated parasites based on size and morphology [14-17].

To develop a more handy plastination technique for *Toxocara* (*Neoascaris*) *vitulorum*, the impregnation of the melamine polymer was performed at normal environmental pressure contrary to the low pressure (below 5 mmHg) need of the conventional silicone plastination technique [14,17,23]. The temperature during plastination was maintained at −20°C. This ultra-low temperature caused fixation and expansion in the specimens and prevents its decomposition [24].

The main purpose of using compounds (e.g., turpentine or clove oil, chloroform, isopropanol, benzene, and xylene) other than melamine polymer was to reduce the shrinkage and to increase the flexibility in the plastinated parasites. These compounds can be used as a plasticizing agent, solvent for thinning paints and varnishes, as a cleaning/clearing agent, cooling agent, etc. The high solvency of xylene/chloroform rendered transparent tissue and enhanced infiltration of the polymer can be observed [25]. Evaluation score for shrinkage and flexibility stands in favor of parasites plastinated in the MTCl solutions, while maximum shrinkage and minimal flexibility were observed in MTB/MTI solution. The shrinkage and flexibility are two major limitations of this technique, especially in the biological samples made up of soft tissues. These limitations were highlighted by Sagoo and Adds [26] in the plastinated brain slice using Biodur TM S10/S3 polymer and Kumar *et al*. [17] in plastinated macroparasites using melamine polymer. As per Latorre *et al*. [11], shrinkage and color changes were the major causes of failures of the plastination technique. The plastinated items stand intact in the environmental condition. It can be presumed that plastinating solution inhibited the growth of the microbes by resisting the entry of water molecules inside the specimens [27].

## Conclusion

Plastination technique can be used as an alternative method of liquid preservation. Plastinated *Toxocara* (*Neoascaris*) *vitulorum* in MTCl has considerable level of flexibility and minimal shrinkage than the parasites plastinated in other polymer solutions. Polymer solution of MTB/MTI caused maximum shrinkage and plastinated roundworm becomes brittle in nature, so their further use should be avoided. There is still a scope of some suitable plasticizing agents to increase the flexibility in the plastinated specimens.

## Authors’ Contributions

NK planned and accomplished the overall research work. DCP and PS extended their physical support in performing the plastination technique. NK did the data analysis, drafted, and revised the manuscript. JBS did the initial revision of the manuscript. RM and SC had extended the technical help in doing the plastination technique. All authors read and approved the final manuscript.
